# Supplementary material for: Traumatic and Diabetic Schwann Cell Demyelination Is Triggered by a Transient Mitochondrial Calcium Release through Voltage Dependent Anion Channel 1
Source: Biomedicines. 2022 Jun 19;10(6):1447. doi: 10.3390/biomedicines10061447 (PMC9220872; doi:10.3390/biomedicines10061447)
Supplement: Supplementary file 1 [file biomedicines-10-01447-s001.zip › biomedicines-1747424-supplementary.pdf]

FIGURE S1

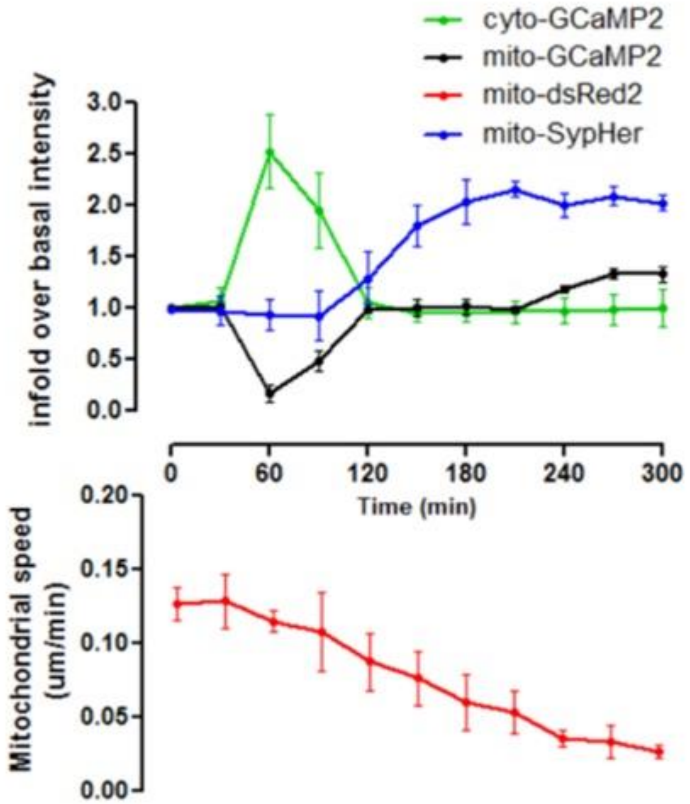

Figure S1. Succession of events occurring in myelinating SC when Wallerian demyelination is triggered

We plotted the dynamics of mito-GCaMP2, cyto-GCaMP2, mito-Sypher probes fluorescence and mito-Dsred2 labelled mitochondria velocity on the same time frame. This shows that the first event to occur is the release of calcium from the mitochondria to the cytoplasm (between 30 and 120 min). At the same time mitochondrial velocity starts to drop regularly to reach immobility by around 300 min. Mitochondrial pH increases (>120 min) to reach a plateau by around 180 min. Then mitochondrial calcium increases (>180 min) reaching a plateau by around 270 min.

FIGURE S2

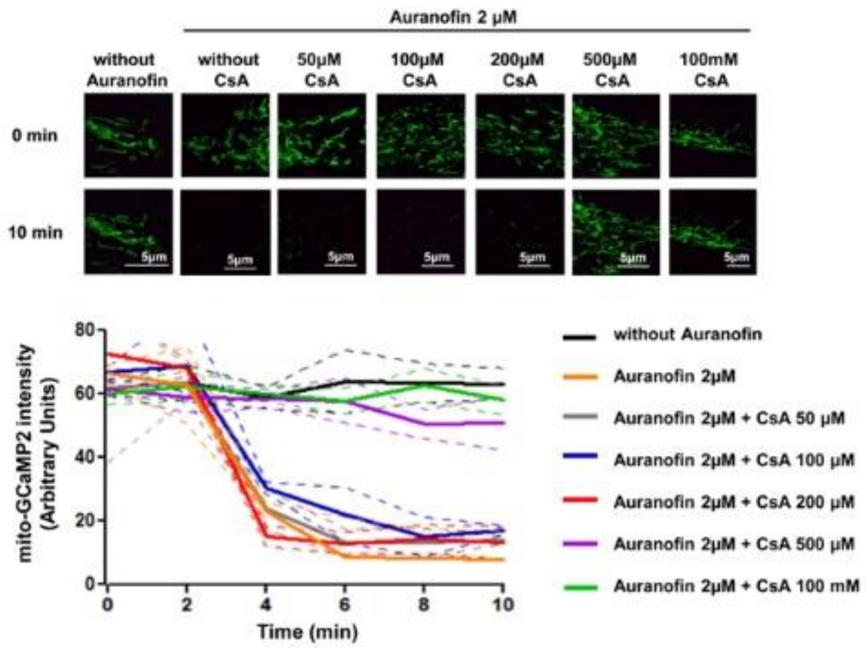

Figure S2. 500 µM cyclosporine A blocks mPTP opening induced by 2µM auranofin in mSC *in vivo*

Mice expressing mito-GCaMP2 in mSC were anesthetized and mitochondrial calcium dynamic was analyzed through live two-photon imaging. Cyclosporine A (CsA) was injected in the nerve 30 minutes before imaging. Then, 2 µm auranofin was also injected in order to open mPTP and to release mitochondrial calcium. When concentration is too low (50, 100 and 200µM) CsA does not prevent mPTP opening with auranofin and mitoCGaMP2 probe fluorescence remains unchanged. Above 500µM CsA blocks mPTP opening despite auranofin inhibition.

Upper panel. Representative images of the mito-GCaMP2 probe fluorescence in the different conditions.

Lower panel. The mean mito-GCaMP2 fluorescence intensity was measured for more than 100 mitochondria at different time points for each conditions. n=2 mice. Dashed lines show the error bars for each condition and each time point.

FIGURE S3

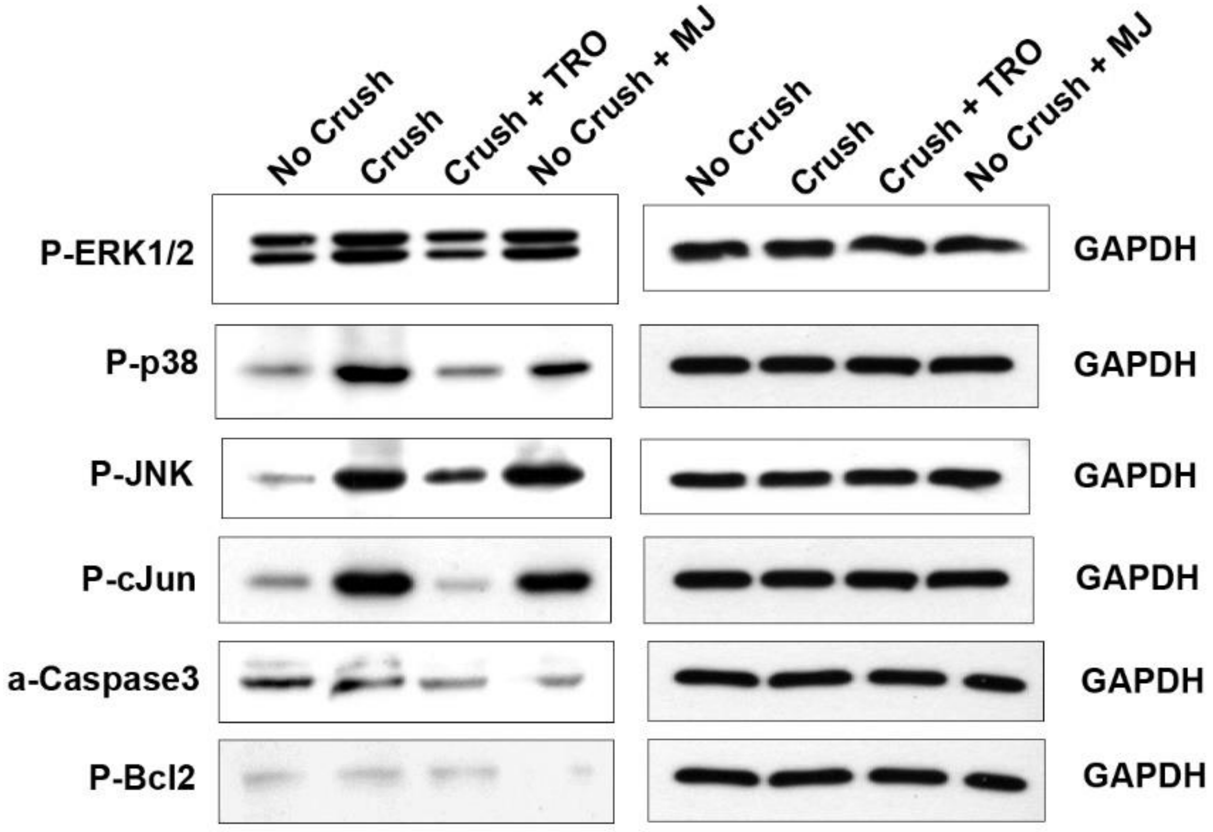

Figure S3. Activation of the demyelination pathways 12 hrs after injury

Western blot analysis for phospho-ERK1/2 (P-ERK1/2), phospho-p38 (P-p38), phospho-JNK (P-JNK), phospho-cJun (P-cJun), activated cleaved-caspase3 (a-caspase3)and phospho-Bcl2 (P-Bcl2) in sciatic nerve of mice without injury (No crush), 12 hrs after injury (Crush), 12 hrs after injury with TRO19622 treatment 30 min before injury (Crush + TRO) or without injury but 12 hrs after methyl jasmonate treatment (No crush + MJ). GAPDH was used as loading control. n= 3 independent experiments.
